# DXA Android-to-Gynoid Ratio and Cardiovascular Risk Assessment in Age and BMI Propensity-Matched Early Postmenopausal Women

**DOI:** 10.3390/medicina60071096

**Published:** 2024-07-04

**Authors:** Irina Manuela Nistor, Simona Fica, Sorina Carmen Martin, Theodor Mustata, Theodor Eugen Oprea, Anca Elena Sirbu, Carmen Gabriela Barbu

**Affiliations:** 1 Department of Endocrinology, “Carol Davila” University of Medicine and Pharmacy, 020021 Bucharest, Romania; 2 Department of Endocrinology, “Elias” University and Emergency Hospital, 011461 Bucharest, Romania; 3 Department of Endocrinology and Metabolism, The National Institute of Endocrinology “CI Parhon”, 011863 Bucharest, Romania

**Keywords:** early menopause, DXA body composition parameters, android fat, gynoid fat, cardiovascular risk, 10-year ASCVD risk

## Abstract

*Background and Objectives*: The literature suggests that physiological menopause (MP) seems linked with increased adiposity with a preference for intra-abdominal fat accumulation, greater than what can be attributed only by aging, which could magnify this period’s increased cardiovascular risk. *Materials and Methods*: We retrospectively analyzed two age and body mass index (BMI) propensity-matched subgroups each formed of 90 clinically healthy, 40–60-year-old postmenopausal women, within the first 5 and 5–10 years of MP. The 10-year ASCVD risk was assessed using medical history, anthropometric data, and lipid profile blood tests. The android-to-gynoid (A/G) ratio was computed using Lunar osteodensitometry lumbar spine and hip scans. *Results*: The A/G ratio was significantly higher for the subgroup evaluated in years 5–10 of MP than in the first 5 years of MP, even after controlling for BMI (1.05 vs. 0.99, *p* = 0.005). While displaying a significant negative correlation with HDL cholesterol (r = 0.406), the A/G ratio also had positive correlations with systolic blood pressure (BP) values (r = 0.273), triglycerides (r = 0.367), and 10-year ASCVD risk (r = 0.277). After adjusting for smoking, hypertension treatment, and type 2 diabetes, the 10-year ASCVD risk became significantly different for women in the first 5 years (3.28%) compared to those in years 5–10 of MP (3.74%), *p* = 0.047. *Conclusions*: In women with similar age and BMI, the A/G ratio appears to vary based on the number of years since menopause onset and correlates with either independent cardiovascular risk parameters like BP, triglycerides, and HDL cholesterol or with composite scores, such as 10-year ASCVD risk.

## 1. Introduction

The body mass index (BMI), calculated as weight (in kilograms) divided by square height (in meters), estimates a person’s body fat and is commonly used to define a person as normal weight, overweight, or obese [1]. Assessing abdominal obesity through the measurement of the waist or hip circumference, as well as the waist-to-hip ratio, provides a more accurate depiction of obesity-related comorbidities, such as cardiovascular diseases (CVD), type 2 diabetes mellitus (T2DM), and metabolic syndrome. The visceral distribution of adipocytes can partially quantify abdominal fat, which functions as an endocrine organ and produces various adipokines and substances associated with insulin resistance, metabolic syndrome, and T2DM. This distribution also contributes to increased inflammation, a significant risk factor for the aforementioned conditions [1,2,3,4]. 

Still, the precision and accuracy of these anthropometric measurements are low compared to imaging, such as dual-energy X-ray absorptiometry (DXA), computed tomography (CT), and magnetic resonance imaging (MRI) [5]. These more expensive and time-consuming techniques can provide precise measurement of subcutaneous adipose tissue (SAT), visceral adipose tissue (VAT), or assessment of fat and lean mass by body region [6,7]. Used for bone mineral density assessment and osteoporosis diagnosis, a simple DXA hip scan provides the percentages of total body fat (TBF%) and android (AF%) and gynoid fat (GF%), which could be useful in the absence of a more time-consuming scan, as the android-to-gynoid (A/G) ratio was correlated with VAT [8]. Android adiposity is defined by intra-abdominal fat, while gynoid adiposity is defined by the accumulation of subcutaneous fat and is characteristic of females [9]. It is presumed that leg fat mass, together with a gynoid type of fat distribution, has a protective association for CVD risk markers, for a better lipid profile [10], and decreased odds of elevated blood glucose [11], while the android fat distribution is associated with insulin resistance [12]. Besides DXA, there are other pieces of equipment with generalized appliance in estimating body composition, such as InBody devices, which use bioelectrical impedance analysis (BIA). Despite concerns that this technique can underestimate body fat, overlooking the distribution of fat and muscle, the advancement of technology and the widespread use of BIA equipment can explain the similar scan results obtained from small studies that compared it to the gold standard, the DXA scan [13,14].

Previous studies suggested that a higher BMI was correlated with an older age of menopause onset in different ethnicities, as higher leptin levels, adipokines produced by the adipose tissue, have a peripheral ovarian action or a central hypothalamic one, augmenting female reproductive hormones [15]. The relationship between adipose tissue and menopause does not end there, as several potential factors, including aging, a decreased metabolic rate due to sarcopenia and decreased physical activity levels, and the hormonal changes from the menopausal transition, have been considered to explain the widely described accumulation of fat during a woman’s midlife period [3]. Menopause leads to a decline in basal energy consumption and a decrease in the appetite-suppressing action of estrogen, which occurs through the stimulation of alpha-receptors in the central nervous system. In order to counteract this predisposition to weight gain, many experts believe that for a healthy menopausal transition, it is necessary to make some behavioral modification, such as reducing calorie intake and engaging in regular physical exercise [16]. Some insights in the topic of fat redistribution associated with menopause were provided by Tremolliere in 1996, who noted a shifting of the fat mass to an upper body location even when total body fat remained unchanged in postmenopausal women, suggesting that the android adiposity may be influenced by menopause more than age alone [17]. Later, two other papers reiterated that abdominal fat redistribution is associated with menopause [18,19]. Even though these data do not necessarily support causality between menopause and abdominal fat, they may provide some explanation for the shift in the cardiovascular profile that occurs in women after ovarian senescence.

The 10-year atherosclerotic cardiovascular disease (ASCVD) risk calculator was derived from the Pooled Cohort Equation published in 2013 by the American College of Cardiology and American Heart Association (ACC/AHA), along with new statin therapy guidelines [20]. Although it was the source of several controversies, this calculator was externally validated in other US-based populations. It focuses on hard clinical outcomes such as heart attack, ischemic stroke, or death from these conditions, and it takes into account age, sex, race, total and high-density lipoprotein-cholesterol (HDL-chol) levels, and treatment for hypertension (HTN), as well as systolic blood pressure (BP), the presence of diabetes, and smoking status as risk factors in the prediction model [21].

This study aims to investigate the possible differences in hip DXA scan-derived composition parameters in age and BMI propensity-matched women in different stages of menopause and their association with cardiometabolic risk factors, respectively, using the 10-year ASCVD risk score.

## 2. Materials and Methods

A total of 1271 consecutive DXA scans of clinically healthy women aged 40–60 acquired between December 2018 and July 2023 at “Elias” University and Emergency Hospital, Bucharest, were retrospectively analyzed. The exclusion criteria consisted of menopause duration for more than 10 years (N = 146), final menstrual period (FMP) before the age of 40 (N = 85), glucocorticoid exposure (N = 60), hypogonadism (N = 17), rheumatoid arthritis (N = 7), and insufficient data (N = 467). For 90 women in the first 5 years of menopause, using propensity score matching with a caliper of 0.01, we were able to identify 90 controls that were scanned during years 5–10 of menopause, which were matched for age and BMI.

All the participants signed an informed consent that allowed for the use of their medical records for scientific research. This study received approval from the hospital Ethics Board, and the planning, data collection, analysis, and reporting were performed according to the 2013 Helsinki Declaration.

Clinical medical data were collected, including age, menarche, age of menopause, height, weight, and systolic and diastolic blood pressure (BP). The last two variables were used to compute the body mass index (BMI). Additionally, we considered factors such as smoking status; detailed histories of diseases like T2DM, hypertension (HTN), and dyslipidemia; and medication usage (statin treatment, HTN treatment, and T2DM treatment). The blood sample was obtained in the morning after an overnight fast, and using the Abbott Architect 8000C Chemistry Analyzer, we collected the total cholesterol, high-density lipoprotein (HDL)-cholesterol, low-density lipoprotein (LDL)-cholesterol, and cholesterol levels. The 10-year ASCVD risk score was calculated using the online MD + CALC 2013 ASCVD Risk Calculator from the AHA/ACC [22], based on the Pooled Cohort Equations [20]. A single osteodensitometry machine (iDXA Prodigy GE Lunar) was used by a single, qualified operator to perform all the scans, following the standard quality control protocol. Using the lumbar spine and hip DXA scan, we collected the android (AF%) and gynoid (GF%) fat percentages and computed the android/gynoid ratio by dividing the AF% by the GF%.

All of this study’s analysis, as well as the propensity score matching, was conducted using IBM SPSS Statistics Version 26, while a *p*-value of less than 0.05 was used to establish statistical significance. The Chi-square test was used to assess the similarity in the percentages of the categorical variables across the subgroups. The Shapiro–Wilk test evaluated the normality of the continuous variables’ distribution. We used the mean ± SD for variables with a normal distribution and the median (interquartile range) for those with a non-normal distribution. The Mann–Whitney U test was utilized to compare the continuous skewed variables between subgroups, whereas the Independent-Samples T Test was used for variables with a normal distribution. To evaluate the association between variables, we used Spearman’s correlation coefficients. A General Linear Model (one-way ANCOVA) was conducted to compare the following variables between the two subgroups (first 5 years of menopause and years 5–10 of menopause): the A/G ratio values while controlling for BMI, total cholesterol levels after adjusting for active smoking status and statin treatment, and 10-year ASCVD risk after adjusting for active smoking status, HTN treatment, and the presence of T2DM diagnosis. Levene’s test and normality checks were conducted, and the assumptions were met.

## 3. Results

Our study group consisted of two subgroups of 90 women each, divided by the period in which they were evaluated from the FMP (first 5 years of MP and years 5–10 of MP). For the whole study group, the mean age at evaluation was 53.36 ± 2.52 years, the mean BMI was 27.97 ± 3.96 kg/m^2^, and the median age at FMP was 48 (4) years old. The two subgroups were propensity score-matched for age and BMI, as seen in Table 1, and showed similar percentages of surgically induced menopause (14/90–15.6% in the first 5 years of MP vs. 20/90–22.2% in years 5–10 of MP, *p* = 0.367) and antiestrogen treatment (7/90–7.77% patients in each subgroup, *p* = 1.000).

**Figure 1:**
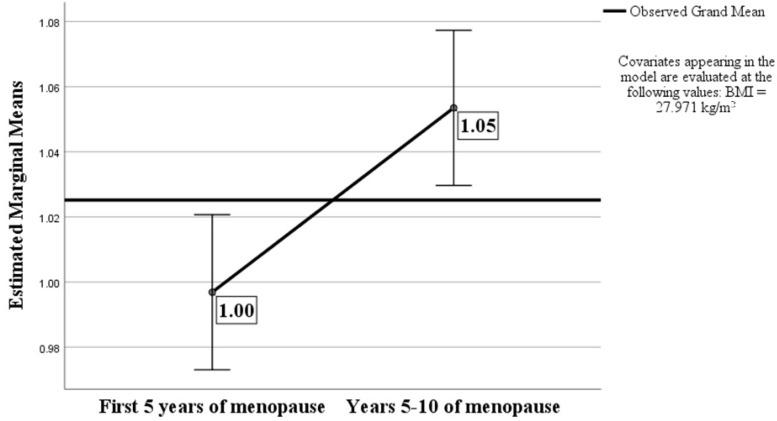
Estimated marginal means of A/G ratio.

The A/G ratio was correlated with the BMI (*p* < 0.001 and r = 0.420), total body fat percentage (*p* < 0.001 and r = 0.426), android fat mass percentage (*p* < 0.001 and r = 0.835), and weight (*p* < 0.001 and r = 0.368). Significant differences in the A/G ratio values were seen between the subjects evaluated during the first 5 years (mean value of A/G ratio of 0.99) vs. years 5–10 of MP (mean value of A/G ratio of 1.05), even after adjusting for a mean value of BMI of 27.97 kg/m^2^, *F*(1, 180) = 11.001, *p* = 0.005, and partial ƞ^2^ = 0.059 (Figure 1).

There were statistically more smokers in the first 5 years of MP compared to the second subgroup (29/90–30% vs. 14/90–15.6%, *p* = 0.032). Despite statistically higher values of systolic BP in years 5–10 of MP compared to first the 5 years of MP (134.04 ± 6.34 mmHg vs. 130.69 ± 7.05, *p* = 0.001), there were no statistically different percentages of HTN treatment, T2DM diagnosis, or statin treatment between the two subgroups, as seen in Table 2.

Although the serum total cholesterol levels were significantly higher in the subgroup with more years of estrogen deprivation, after adjusting for active smoking status and statin treatment they did not maintain their statistical difference between the two subgroups: 215.54 mg/dL for the first 5 years of menopause and 223.37 mg/dL for years 5–10 of menopause, *F*(1, 164) = 1.254, *p* = 0.264, and partial ƞ^2^ = 0.008.

**Figure 2:**
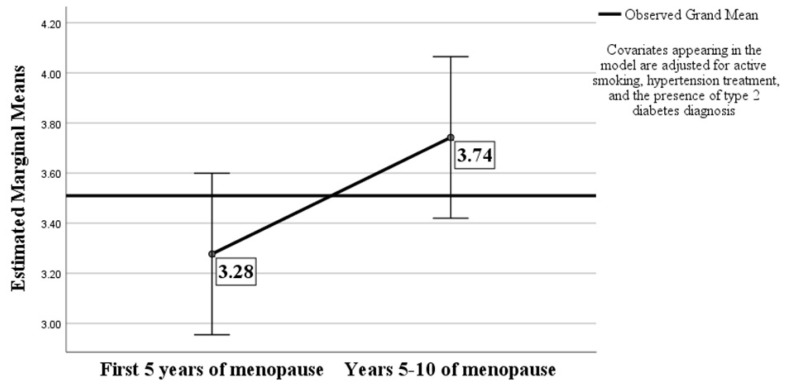
Estimated marginal means of 10-year ASCVD risk score.

The 10-year ASCVD risk was correlated with age (*p* < 0.001 and r = 0.353), menopause age (*p* < 0.001 and r = 0.291), BMI (*p* < 0.001 and r = 0.288), systolic BP values (*p* = 0.015 and r = 0.194), HDL-cholesterol values (*p* < 0.001 and r = −0.456), triglyceride levels (*p* < 0.001 and r = 0.469), smoking status (*p* < 0.001 and r = 0.622), hypertension treatment (*p* < 0.001 and r = 0.423), and the presence of diabetes mellitus diagnosis (*p* < 0.001 and r = 0.364). After adjusting for smoking status, HTN treatment, and the presence of T2DM, the 10-year ASCVD risk became statistically different between the subjects in the first 5 years of menopause (3.28%) vs. years 5–10 of menopause (3.74%): *F*(1:158) = 3.993, *p* = 0.047, and partial ƞ^2^ = 0.025–Figure 2.

In our study group, besides the positive correlation with the body fat parameters, the A/G ratio was also associated with systolic BP values (*p* = 0.003 and r = 0.237), HTN treatment (*p* = 0.003 and r = 0.217), triglyceride levels (*p* < 0.001 and r = 0.367), and 10-year ASCVD risk (*p* < 0.001 and r = 0.277) while displaying a negative correlation with HDL cholesterol (*p* < 0.001 and r = −0.406).

## 4. Discussion

The menopause onset age was 48 years old in our study group, approximately three years earlier than the median European age reported as 50.1–52.8 years old [23]. Not only weight but also genetic and demographic peculiarities, along with the aging process of the neuroendocrine system via interactions of multiple genes [24,25], can influence the natural timing of menopause [26]. Lifestyle and reproductive characteristics, general health status, or even the intrauterine period—the prenatally determined size of the ovarian pool—can damage the oocytes and, therefore, influence reproductive aging [27,28,29]. The younger age of menopause compared to the literature might be due to the small number of subjects or to an ethnic characteristic, as another Romanian study that assessed the quality of life of 364 postmenopausal women with osteoporosis and fragility fractures reported a similar mean age of 48 years old for menopause onset [30].

The continuous decline in endogenous estrogen levels is considered by some studies to impact body fat mass and distribution, resulting in an enhanced amount of central fat in postmenopausal women [3,31]. As the change in fat mass quantity between pre- and postmenopausal women was explained by aging theory with no significant additional influence of menopause per se, the studies are controversial in declaring whether menopause or age, as ovarian aging is simultaneous with chronological aging, influences fat distribution in contrast to body weight or BMI [3,12,32,33,34]. Our age- and BMI-matched study’s results state that women in years 5–10 of MP had a statistically higher value of the android-to-gynoid ratio compared to those in the first 5 years of menopause. Previous studies indicated that the onset time of abdominal fat accumulation was early postmenopause, in which the ovaries were hormonally active for secreting testosterone [18,35]. Not only do changes in adiposity foreshadow changes in sex steroids during the menopausal transition, as waist circumferences predicted future levels of estradiol, testosterone, follicle-stimulating hormone (FSH), and SHBG, but sex steroids and FSH can also cause a change in fat distribution [4]. Visceral fat expresses FSH receptors, and fat mass redistribution was observed in mice after in vitro administration of FSH to preadipocytes, which increased adipocyte lipid synthesis [4].

A preceding study from 1996 that included 68 premenopausal women (mean age 49.3 ± 3.2 and mean BMI 21.6±) and 100 women in early postmenopause (less than 60 years old; mean age 53.8 ± 3.1, mean BMI 21.8 ± 1.8, and mean years since MP 3.3 ± 2.2) found that the distribution of android fat was greater in the young postmenopausal women (48.3 ± 4.4%) compared to the premenopausal subjects (46.2 ± 4.4%), *p* < 0.001, although the absolute amount of body fat mass did not significantly differ between the two subgroups; also, the percentage of android fat increased both with age and with years since MP, as a significantly higher proportion of android fat was found in postmenopausal women within 3 years of menopause, compared to premenopausal women. The study’s authors considered that, despite the slight age difference between the two subgroups, there is a menopause effect on fat distribution rather than an age effect [17]. In our study, the A/G ratio did not correlate with age or with years since FMP, but we consider this result to be the consequence of the extremely specific and limited age frame of our early postmenopausal subjects.

A 2013 study that included 105 early postmenopausal women (with less than 5 years of MP) and 107 late postmenopausal women (more than 10 years of MP) found that the latter had a higher percentage of body fat compared with the former (34.86 ± 4.29% vs. 33.05 ± 3.50%; *p* < 0.01) but with no significant differences in body fat distribution, BMI, or waist-to-hip ratio between the subgroups. Only in obese or overweight women did body fat have a greater tendency to accumulate in the abdomen compared to normal-weight women in both subgroups [35]. Research data have shown that not only do women who are obese have a higher risk of hypertension, dyslipidemia, and a 4-fold increase in cardiovascular death rate [2], but the central fat distribution in women with a BMI of 18.5 to 25 kg/m^2^ increases the mortality risk as high as in overweight women with central obesity [36]. Obesity and insulin resistance were statistically associated with the risk of incident hypertension in healthy, non-diabetic, recently postmenopausal Greek women with normal renal function, with a median time to the diagnosis of incident hypertension of 3.5 years [37]. Studies of 27 populations from Europe, Australia, New Zealand, and Canada showed that dyslipidemia was strongly associated with a higher waist circumference in postmenopausal women after adjusting for region, age, and smoking [38].

There is limited knowledge about the correlation between the android-to-gynoid ratio and 10-year ASCVD risk, as a major study based on the NHANES 2003–2007 cohort only discussed the individual relationships between cardiovascular mortality in adults and android or gynoid fat mass percentages, as acquired from a whole body scan performed on a Hologic DXA machine [39]. Although the study showed that increased gynoid fat mass was a protective factor for all-cause mortality in older females, the people with more android and gynoid fat mass from the study had a lower risk of cardiovascular disease in the absence of high BP. The comparison with our study seems ill-advised, as their analysis relied on absolute values and their corresponding quartiles, obtained from another type of osteodensitometry machine.

According to another study that included 685 normal weight women (mean age 35.1 ± 15) from the NHANES 2005–2006 cohort [11], the investigated cardiometabolic risk factors showed a less strong correlation with BMI than with the A/G ratio, even in normal BMI American women. In their study group, the A/G ratio was positively correlated with triglycerides (r = 0.370), similar to our findings (r = 0.367, *p* < 0.001), and systolic blood pressure (r = 0.203 in the American study vs. r = 0.237, *p* = 0.003 in our study group) and was negatively correlated with HDL-cholesterol levels (r = −0.384 in the NHANES cohort vs. r = −0.406, *p* < 0.001 in our study group). Dehghan et al. [12] declared that body composition changes and increases in VAT do not significantly change the degree of the well-established cardiovascular risk from the menopause transition period, as a number of effects suggest that perimenopausal qualitative and functional changes in end organs, such as adipose tissue and liver, could have a stronger impact on cardiovascular risk than changes in body composition. However, Aggarwal et al. [40] showed that body fat percentage was associated with dyslipidemia in perimenopausal women. In our study group, the A/G ratio showed small, but significant correlations not only with triglyceride and HDL-cholesterol levels but also with 10-year ASCVD risk scores. As studies suggested that increases in total body fat should be of interest across the lifespan, attention should be paid to the accumulation of central fat and the decrease in total leg fat after menopause [3].

We considered it essential to acknowledge the small number of patients as a study limitation, as well as the lack of trustworthy anthropometric data that could have evaluated the relationship between this study’s parameters and the waist-to-hip ratio. Also, the lack of former smoker and aspirin treatment data for all the participants led to an inability to use the ASCVD Risk Estimator Plus calculator. Given that changes in regional fat are commonly linked to weight gain and physiological aging, we deemed it necessary to utilize propensity score matching for age and BMI to detect subtle variations in the fat distribution. Therefore, although we did not expect to observe striking differences in these parameters, even a subtle variation in the regional fat distribution can be considered a strength point, considering this study’s design. While we noticed a notable difference in the A/G ratio among the subgroups, it is worth mentioning that all the women participating in this study are relatively young and in the early stages of menopause. The relationship between the years since menopause and the A/G ratio at similar ages can be further defined by a larger-scale comparison with women with more years of estrogen deprivation. However, this approach presents recruitment issues and the potential for bias due to ovarian insufficiency or other secondary amenorrhea disorders, which could impact the results. As our subgroups were matched for age, the shorter period of estrogen exposure due to the earlier menopause onset for the patients evaluated during years 5–10 of menopause could contribute to a poorer cardiovascular risk. We wish to address the relationship between cardiovascular risk and the years of estrogen exposure in further studies. Another limitation of the present study is the lack of data from the fertile period of our patients that could count as possible protective factors, such as number of pregnancies and time spent breastfeeding.

As a whole-body DXA scan is more time consuming and could be seen as an unnecessary radiation exposure in the absence of a more deserving clinical setting, studies should confirm the possible usage of android and gynoid fat percentages derived from the hip DXA scan, a usual procedure in the assessment of osteoporosis after menopause, as corresponding markers for their equivalent body composition parameters from the total body scan. Moreso, the relationship between these parameters and the cardiovascular risk factor, expressed individually or compiled into a risk score such as the 10-year ASCVD, can be useful in leading the clinical and paraclinical evaluation of clinically healthy, early postmenopausal women. A potential clinical usage of our obtained results is the long-term benefit due to lifestyle and dietary modifications for young postmenopausal women with poorer cardiovascular prediction and a higher android-to-gynoid ratio, commonly screened for osteoporosis.

## 5. Conclusions

In our age and BMI propensity-matched, clinically healthy young postmenopausal women, the android-to-gynoid ratio was significantly higher in the subjects within years 5–10 of menopause compared to those in the first 5 years of menopause, even after further adjusting for BMI. The android-to-gynoid ratio was negatively correlated with HDL-cholesterol levels and positively with systolic blood pressure values, triglyceride levels, and 10-year ASCVD risk. After controlling for smoking status, hypertension treatment, and the presence of type 2 diabetes diagnosis, the 10-year ASCVD risk was also significantly higher in women evaluated in years 5–10 of menopause compared to those in the first 5 years of menopause.

## Figures and Tables

**Table 1:** The general characteristics of the first 5 years and years 5–10 of MP subgroups created using age and BMI propensity score matching (caliper 0.01).

	First 5 Years of MP (N = 90)	Years 5–10 of MP (N = 90)	Sig
Age	53.53 ± 2.56	53.44 ± 2.48	0.811
Age at FMP	50.06 ± 2.63	46.07 ± 2.86	<0.001
Years of Estrogen Exposure	37 (3)	32.5 (4)	<0.001
Years of MP	3.55 (1.8)	7.15 (2.7)	<0.001
BMI (kg/m^2^)	27.79 ± 4.12	28.03 ± 3.83	0.692
Total Body Fat %	45.9 (6.4)	47.25 (7.2)	0.086
Android Fat %	47.8 (10.6)	50 (7.4)	0.180
Gynoid Fat %	46.5 (4.8)	47.3 (6.3)	0.371
A/G Ratio	0.99 ± 0.13	1.05 ± 0.11	0.001

**Table 2:** Cardiovascular risk factor parameters of the two study subgroups (first 5 years and years 5–10 of MP subgroups created using age and BMI propensity score matching).

	First 5 Years of MP (N = 90)	Years 5–10 of MP (N = 90)	Sig
HTN treatment (%)	30/90 (33.3%)	31/90 (35.5%)	1.000
Diagnosis of T2DM (%)	8/90 (8.9%)	6/90 (6.7%)	0.650
Statin treatment (%)	24/90 (26.7%)	20/90 (22.2%)	0.603
Serum total cholesterol (mg/dL)	207 (54)	226 (62)	0.019
Serum HDL cholesterol (mg/dL)	57 (17)	54.5 (15.25)	0.527
Serum LDL cholesterol (mg/dL)	131.6 (46.8)	140.4 (55.1)	0.132
Serum triglycerides (mg/dL)	87 (58)	97 (59.25)	0.214
10-year ASCVD risk (%)	2.95 (2.5)	2.7 (2.41)	0.340
HTN treatment (%)	30/90 (33.3%)	31/90 (35.5%)	1.000

## Data Availability

The data presented in this study are available on request from the corresponding author due to privacy reasons.

## Abbreviations

American College of Cardiology and American Heart Association (ACC/AHA), android fat percentage (AF%), android to gynoid (A/G), atherosclerotic cardiovascular disease (ASCVD), bioelectrical impedance analysis (BIA), blood pressure (BP), body mass index (BMI), cardiovascular disease (CVD), computed tomography (CT), dual-energy X-ray absorptiometry (DXA), final menstrual period (FMP), gynoid fat percentage (GF%), high-density lipoprotein cholesterol (HDL-chol), hypertension (HTN), low-density lipoprotein cholesterol (LDL-chol), magnetic resonance imaging (MRI), menopause (MP), standard deviation (SD), subcutaneous adipose tissue (SAT), total body fat percentage (TBF%), type 2 diabetes mellitus (T2DM), and visceral adipose tissue (VAT).
